# Hybrid Cooperative Cache Based on Temporal Convolutional Networks in Vehicular Edge Network

**DOI:** 10.3390/s23104619

**Published:** 2023-05-10

**Authors:** Honghai Wu, Jichong Jin, Huahong Ma, Ling Xing

**Affiliations:** School of Information Engineering, Henan University of Science and Technology, Luoyang 471000, China; honghai2018@haust.edu.cn (H.W.); 200320050364@stu.haust.edu.cn (J.J.); mhh@haust.edu.cn (H.M.)

**Keywords:** vehicle edge network, cooperative cache, temporal convolutional networks

## Abstract

With the continuous development of intelligent vehicles, people’s demand for services has also rapidly increased, leading to a sharp increase in wireless network traffic. Edge caching, due to its location advantage, can provide more efficient transmission services and become an effective method to solve the above problems. However, the current mainstream caching solutions only consider content popularity to formulate caching strategies, which can easily lead to cache redundancy between edge nodes and lead to low caching efficiency. To solve these problems, we propose a hybrid content value collaborative caching strategy based on temporal convolutional network (called THCS), which achieves mutual collaboration between different edge nodes under limited cache resources, thereby optimizing cache content and reducing content delivery latency. Specifically, the strategy first obtains accurate content popularity through temporal convolutional network (TCN), then comprehensively considers various factors to measure the hybrid content value (HCV) of cached content, and finally uses a dynamic programming algorithm to maximize the overall HCV and make optimal cache decisions. We have obtained the following conclusion through simulation experiments: compared with the benchmark scheme, THCS has improved the cache hit rate by 12.3% and reduced the content transmission delay by 16.7%.

## 1. Introduction

With the rapid development of the Internet of Things (IoT) [1] and Artificial Intelligence (AI) [2] technology, intelligent connected vehicles (ICV) are on the rise. Gartner predicts that in 2023, most of the vehicles on the road will be connected to the Internet, and connected vehicles will be the largest market for 5G in 2025. Traditional vehicle networks are extensions of mobile networks that provide services for a variety of applications such as autonomous driving, traffic management, and driving safety [3]. In addition, most users of autonomous vehicles are also more focused on entertainment services. Emerging services include video, news, road inquiries, and other entertainment and leisure information services, which enhance the convenience and experience of users’ travel. Due to the high popularity of intelligent infotainment services, these contents will be frequently requested, so a large number of users and demand data will bring challenges to the performance of the wireless network, which may greatly affect the user experience.

Edge caching [4,5] is an effective way to cope with these problems. By arranging caching resources and computing resources at the edge servers, various computing and request services for ICVs can be satisfied at the edge servers, thus allowing users to obtain a better quality of experience (QoE). Therefore, edge caching networks for vehicles have recently attracted widespread attention in the academic community. For example, the authors in [6] explore active caching algorithms for high-capacity content. There are also caching schemes that use mobility prediction [7,8] and cooperative caching [9] to reduce network latency and increase network throughput. Most of these works use Zipf distribution models to model the popularity of content. However, in a real vehicle scenario, where the requested content is time-local and variable, modeling popularity using only the Zipf distribution model is not optimal. Therefore, building an efficient edge caching strategy for ICVs is challenging.

To solve the above problem, we formalize the cooperative caching process between edge nodes as an optimization problem, using our proposed cooperative caching algorithm to allocate edge cache resources reasonably to minimize the latency of content delivery. For this purpose, we creatively propose a hybrid cooperative caching strategy based on temporal convolutional networks, which mainly consists of three parts. Firstly, according to the mobility of vehicles, a dynamic vehicle cluster is established in this paper, then we select the cluster head (CH) and obtain the optimal cache location through the CH. Secondly, we have constructed a model that can improve the accuracy of content popularity prediction, which is based on a temporal convolutional network. TCN has the advantages of high parallelism and fast convergence speed. Then, considering that there are other factors that affect the caching performance of edge nodes, we use Hybrid Content Value to measure the value of the cached content. Finally, a dynamic programming scheme is designed to optimize the strategy. Our main contributions are as follows:

- We predict content popularity based on TCN, and then a hybrid content value is proposed to measure the cached content;
- Dynamic programming is proposed to make the best cache decision and maximize the overall HCV of the cache strategy;
- Simulation experiments verify the excellent caching performance of THCS algorithm.

The structure of the remaining parts is as follows. In Section 2, we discuss related work. Section 3 briefly introduces the system model. In Section 4, we detail the design of the THCS scheme and then propose dynamic programming for caching decisions. The performance of our proposed THCS strategy is verified by simulation in Section 5 and then summarized in Section 6.

## 2. Related Works

A great deal of work has been focused on the study of caching policies. In this section, we present some of the current work in caching research, mainly covering both non-cooperative and cooperative caching.

### 2.1. Non-Cooperative Caching Strategy

In recent years, edge caching has played an important role in in-vehicle networks. Many existing articles design caching strategies by exploiting various properties of the content. For example, Ostrovskaya et al. [10] proposed a caching strategy for vehicular named data networks. The strategy considers three metrics, freshness of the content, popularity and the distance between the cache location and the cache’s current location. Yao et al. [11] uncover the relevance of content and space by investigating various areas in a city. Edge caching strategies in the Internet of Vehicles (IoV) can also be designed by analyzing the traffic characteristics of roads; modeling the mobility of vehicles has been introduced in models that consider the quality of service of caching. Al Nagar et al. [7] considered the effect of vehicle speed on the optimal caching decision and obtained the gain of content-active caching in roadside units (RSUs) to find the optimal caching strategy. Graph neural network [12] is a deep learning method which can deal with graph structure data and can represent many complex relationships. The graph neural network in the Internet of Vehicles can use the dynamic topological relationship between vehicles to learn the characteristics and behavior of vehicles, so as to perform tasks such as prediction, control, and optimization and realize real-time information update and transmission. Lian et al. [13] proposed a mobile edge caching strategy based on the spatiotemporal graph convolution model (STCC) by mining the spatiotemporal features of content popularity and then using heuristic strategies to minimize content access delays. Zhou et al. [14] designed a caching decision based on the simplex algorithm based on the prediction of the Spatio-Temporal Graph Neural Network (STGNN) and performed edge computing on the edge server in the 6G Internet of Vehicles to achieve rapid response to delay-sensitive tasks. Zhang et al. [15] developed a caching model to optimize network energy efficiency by using mobile vehicles on the edge. Through nonlinear technology and Lyapunov optimization theory, an online caching scheme was proposed to minimize network energy consumption, but the effect of cache file size was ignored. Kong et al. [16] proposed a security query scheme for the distribution of vehicle fog data. Reversible matrix is used to construct data requests from different vehicles to break the relationship between specific data requests and their original vehicles. This scheme significantly reduces the communication overhead on the premise of ensuring the security goal. Based on the problem of a large number of short video files, Song et al. [17] proposed a cache scheme considering the QoE of vehicle Internet users and optimized the cache content by establishing a user interest class model. In [18], in order to solve the delay-sensitive service, the authors jointly considered communication, caching, and computing to optimize the cache content retrieval delay, but they ignored the selection process of cache content when considering cache. Although these studies have made contributions to edge caching, most of them do not take into account the cooperation between edge nodes, which can easily cause the problem of cache redundancy, greatly waste edge storage space, and make it difficult to provide good cache services.

### 2.2. Cooperative Caching Strategy

As different caching strategies are proposed, caching research considering the cooperation between different nodes has gradually become the mainstream research direction. Amer et al. [19] proposed a cooperative cache architecture between clusters in order to improve communication quality and network performance, and optimized the network average delay by greedy algorithm. Wu et al. [20] proposed a user-centered content transmission scheme to cope with the growing number of video files in cellular networks, which improves the QoE of users by sharing cached content among cooperative users, but they ignore the communication interference between heterogeneous users. Ma et al. [21] modeled pre-caching and task allocation as a Markov Decision Process and obtained the optimal proportion of allocation among cooperative nodes through DDPG, so as to improve the data reception rate of mobile vehicles. Zhang et al. [22] responded to the dynamic changes of caching strategy in IoV with the network environment through cooperative caching in two-layer heterogeneous networks. Based on the random geometry theory, the analysis framework of spatial cooperative caching strategy is established, which effectively reduces the network load and provides better quality of service (QoS). Yu et al. [23] proposed a cooperative cache based on federated learning and considering vehicle mobility, which uses the deep learning model deployed by federated learning on mobile vehicles to predict regional content popularity, which protects user data security and improves caching efficiency, but the autopilot scenario they considered is not suitable for reality. Zhu et al. [24] proposed multi-layer cooperative caching in terrestrial satellite integrated network to reduce communication delay and optimized cache performance through cooperative and non-cooperative caching strategies. Rottenstreich et al. [25] introduced the dependency relationship between storage items in cooperative cache and put forward the problem of cooperative cache with dependency, which laid the foundation for cooperative cache with dependency. Chang et al. [26] studied the problem of cooperative edge caching in foggy wireless access networks (F-RAN). The cooperation between DDQN and Fog access Point (F-AP) is applied to formulate the global optimal caching strategy. Yao et al. [27] proposed the problem of cooperative caching in vehicle content centers, which comprehensively considered the future location of edge nodes and the cache redundancy between cooperative nodes to formulate a replacement strategy. Yang et al. [28] studied the multi-hop cooperative caching of wireless sensor networks on ICN and made a trade-off between saving energy and reducing delay. Although the above work takes into account the cooperation of nodes to optimize the cache strategy, most of them only consider content popularity as a measure of cache content. First of all, this will lead to cache redundancy among multiple nodes. Secondly, only considering the content popularity will affect the overall cache performance, resulting in high transmission delay.

## 3. System Model

We introduce the hybrid cooperative cache model of vehicle edge network based on temporal convolution network and then discuss the optimization of transmission latency in different scenarios.

### 3.1. Network Model

**Figure 1:**
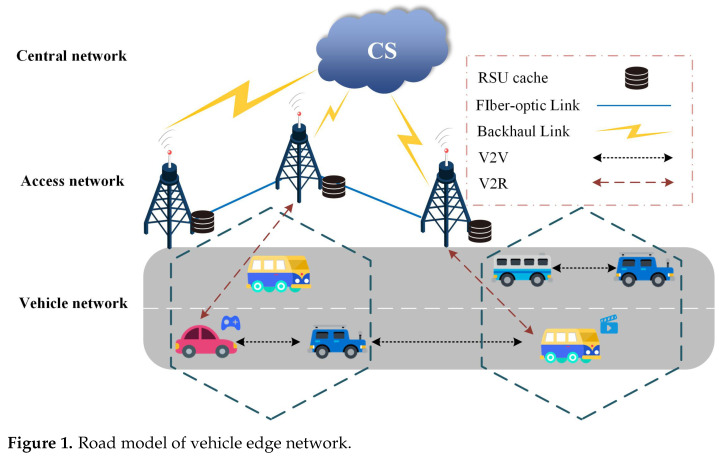
Road model of vehicle edge network.

This paper considers the vehicular network environment based on intelligent transportation system. As shown in Figure 1, the network consists of a central server, RSUs, and various smart vehicles, allowing any edge node to cache content. We assume that the central server stores all the available content in the network, RSUs communicate with each other through optical fiber, and edge servers for computing and caching are deployed, so the RSU can cache all kinds of content to meet the content service needs of vehicle users. Since we have built a dynamic vehicle cluster, when an intelligent vehicle issues a content request, the flow of the network system is as follows:

The intelligent vehicle detects whether the requested content is cached by itself and the cluster head of the cluster, and if so, it can be obtained directly and the delay is generally ignored. Otherwise, the intelligent vehicle checks whether the request content is cached in the local RSU, and if so, the local RSU sends the content directly to the user; otherwise, the next step is to find out if there is any requested content in the nearby RSU. Second, the central server finds out whether the collaborative RSU has cached the content. If so, the cooperative RSU sends the requested content to the user through the retransmission of the local RSU, otherwise, the requested content can only be obtained from the central server. The central server forwards the request to the local RSU, which then sends it to the user by the local RSU. It is worth noting that obtaining request content from a central server increases delivery latency and data traffic compared with obtaining it from an RSU or local node. Nevertheless, the cache capacity on local or edge nodes is limited, and user preferences vary. Therefore, it is challenging to design the optimal caching strategy.

### 3.2. Problem Formulation

As shown in Figure 1, it is assumed that *M* RSU servers are deployed in a cooperation area, where the set of RSU servers is $R=\left\{r_{1},r_{2},\ldots ,r_{M}\right\}$, the cache capacity is $U=\left\{u_{1},u_{2},\ldots ,u_{M}\right\}$, and the set of intelligent vehicles is *N*. Let $r_{0}$ denote a central server and $c_{0}$ represent its capacity. By default, the storage capacity of the central server is unlimited. Assume that the central server has *F* content sets of $E=\left\{e_{1},e_{2},\ldots ,e_{F}\right\}$, and the corresponding size of each content is $S=\left\{s_{1},s_{2},\ldots ,s_{F}\right\}$. Each user individually requests content $e_{i}\left(1\leq i\leq F\right)$ from RSUs in the cooperation area. We use $P_{m,i,t}$ to express the popularity of the content $e_{i}$ on RSU $r_{m}$ at time *t*. We assume that content popularity is static during a given time interval. Cached information can be shared among collaborating RSU. We constructed a content cache matrix $X={\left(x_{m,i}\right)}_{M\times F}$.

(1) $$x_{m,i}=\left\{\begin{array}{cc} 1 & \;\mathrm{if}\;\mathrm{content}\;e_{i}\;\mathrm{is}\;\mathrm{cached}\;\mathrm{on}\;r_{m}\\ 0 & \;\mathrm{otherwise}\; \end{array}\right.$$

The user’s request mode for content will not change during the time slot ▵t. Based on this, we designed a cooperative caching strategy to maximize user-perceived QoE. Since content delivery delays are the most critical factor affecting QoE, we chose it as the main indicator of system evaluation. According to the network model of this paper, the content delivery delay is divided into three parts: the content transmission delay from the central server to the RSU, the content sharing delay between the RSU, and the content acquisition delay from the local RSU to the vehicle. We consider that the communication between vehicles and nodes uses the dedicated short-range communication technology based on 802.11 p and the communication between RSU uses wired links. In short, the problem of edge caching translates into how to minimize the overall latency under the constraint of cache capacity.

According to Shannon’s theorem, the transmission rate between cooperative vehicles and RSU in the cluster is calculated as:

(2) $$d_{r,n}=B\log_{2}\left(1+\frac{Ph_{n}\left(\tau \left(x,V_{n}\right)\right)}{\sigma^{2}}\right)$$

where *B* is the transmission bandwidth, *P* is the transmission power, $\sigma^{2}$ is Gaussian white noise, $h_{n}\left(\tau \left(x,V_{n}\right)\right)$ is the channel gain modeling of vehicle *n* [29], *x* represents vehicles, RSU, and central servers.

The transmission delay from RSU $r_{n}$ to RSU $r_{m}$ consists of two parts: (1) transmission delay $d_{r_{n},r_{m}}^{t}$; (2) propagation delay $d_{r_{n},r_{m}}^{p}$. Therefore, the transmission delay $d_{r_{n},r_{m},i}$ for content $e_{i}$ from RSU $r_{n}$ to RSU $r_{m}$ is as follows:

(3) $$d_{r_{n},r_{m},i}=d_{r_{n},r_{m}}^{t}s_{i}+d_{r_{n},r_{m}}^{p}$$

When sending content $e_{i}$ to $r_{m}$, the cooperation RSU with the smallest delivery delay is:

(4) $$r_{\varphi }=\underset{r_{n}}{\arg\min}\left\{d_{r_{n},r_{m},i}|\forall r_{n}\in \left\{R\cup \left\{r_{0}\right\}\right\},x_{n,i}=1\right\}$$

The content delivery delay of the RSU $r_{m}$ acquiring content $e_{i}$ is $d_{r_{\varphi },r_{m},i}$. If the content $e_{i}$ is cached locally on the RSU server $r_{m}$, $r_{\varphi }=r_{m}$, the content delivery delay is $d_{r_{\varphi },r_{m},i}=0$; if the content $e_{i}$ is not cached on $r_{m}$, but on the rest of the RSU within the collaboration scope, the collaborative RSU transmission content with the minimum delivery delay will be selected. In this case, $r_{\varphi }\in R$, the content delivery of the acquisition request is delayed to $d_{r_{\varphi },r_{m},i}$; if the content $e_{i}$ is not cached in any RSU server within the scope of collaboration, but is transferred from the central server to the local RSU and distributed to the user, then $r_{\varphi }\notin R$, the content delivery of the get request is delayed to $d_{r_{0},r_{m},i}$.

Therefore, the optimization goal P1 of cooperative caching is to minimize the average content delivery delay when the RSU cache capacity is limited. The optimization objectives are as follows:

(5) $$\begin{array}{c} P1:\min\sum_{m=1}^{M}\sum_{i=1}^{F}P_{m,i,t}d_{r_{\varphi },r_{m},i}\\ \;s.t.\;\sum_{i=1}^{F}x_{m,i}\;s_{i}\leq u_{m},\forall r_{n}\in R,\\ x_{m,i}\in \left\{0,1\right\},\forall r_{n}\in R,\forall e_{i}\in E,\\ x_{0,i}=1,\forall e_{i}\in E. \end{array}$$

The goal is to minimize content delivery latency. The first constraint ensures that the total amount of content cached in the RSU does not exceed the capacity of the RSU $r_{m}$. The last constraint indicates that the central server stores all the content. The content in this question only has the option of caching, and no content can be partially cached.

## 4. Cooperative Caching Decisions Based on Temporal Convolutional Networks and Hybrid Content Values

In this section, we first construct dynamic vehicle clusters and then propose a cooperative caching strategy (called THCS) based on temporal convolutional networks and hybrid content value to solve the above problem.

### 4.1. Dynamic Cluster Construction

In order to efficiently deliver content and reduce the load of network traffic, this paper divides all nodes in the edge into several clusters. In order to reduce the communication interference within the cluster, the cluster members considered in this paper cannot communicate directly, and any communication can only be carried out through CH. Each cluster consists of cluster heads and cluster members (CM). Each cluster can have multiple CM but only one CH, depending on the coverage of CH and the size limit of the cluster. Each type of node in the cluster has different responsibilities, and CH is mainly responsible for caching all CM updated cache content information. Then, all nodes in the cluster except CH are CM. CM can be cached, and all CM participate in the CH selection process.

#### 4.1.1. Cluster Head Selection

This paper uses the weighted clustering algorithm (WCA) to select cluster heads, while considering different factors in Equation (5). Where the node with the lowest calculated score is selected as CH, the factors considered are as follows:

(1) Specifying the reciprocal of node degree $1/o_{\alpha }$ of node α;

(2) Specifying the average transmission time $T_{\alpha }$ from the node to all neighboring nodes in the node α range;

(3) The weighted sum $H_{\alpha }$ of the neighbor jump distance of node α;

(4) Specifying the cumulative power $P_{\alpha }$ of node α during the time it acts as CH.

If the value of node α is $w_{\alpha }<w_{min}$, it is selected as a CH node and then added to the CH list. This is expressed as follows:

(6) $$w_{\alpha }=w_{1}\frac{1/o_{\alpha }}{{\bar{M}}_{o}}+w_{2}\frac{T_{\alpha }}{\bar{T}}+w_{3}\frac{H_{\alpha }}{\bar{H}}+w_{4}\frac{P_{\alpha }}{\bar{P}}$$

where $w_{1}$, $w_{2}$, $w_{3}$, $w_{4}$ is the weight factor corresponding to different parameters,

(7) $$w_{1}+w_{2}+w_{3}+w_{4}=1$$

$o_{\alpha }$ in Equation (8) is the degree of node α expressed as:

(8) $$o_{\alpha }=\mathrm{Neighbor}\left(\alpha \right)\sum_{q\in N,q\neq n}\mathrm{dist}\left(\alpha ,q\right)\in \left(0,1\right)$$

where dist(α,q)=1 indicates that *q* is within the transmission range of node α, otherwise it is 0. The average reciprocal of the node degree of all network nodes ${\bar{M}}_{o}$ is as follows:

(9) $${\bar{M}}_{o}=\frac{1}{N}\sum_{\alpha =1}^{N}\frac{1}{o_{\alpha }}$$

$T_{\alpha }$ is expressed as follows:

(10) $$T_{\alpha }=\frac{1}{\psi_{\alpha }}\sum_{q\in \psi_{n}}t_{q,\alpha }$$

where $\psi_{\alpha }$ is a collection of nodes α within the scope of the cluster, and $t_{q,\alpha }$ represents the transfer time from node α to any node *q*. Similarly, the average transmission time of all network nodes is:

(11) $$\bar{T}=\frac{1}{N}\sum_{\alpha =1}^{N}T_{\alpha }$$

In order to reduce the communication load, CH should choose nodes with few hops to establish communication. The $H_{\alpha }$ value is as follows:

(12) $$H_{\alpha }=\sum_{k=1}^{\infty }\frac{\gamma_{k}\times k}{\;\mathrm{count}{\;}_{k}}$$

where $count_{k}$ is the number of k−hop neighbors of node α and $\gamma_{k}$ is the weight coefficient. The average weighted sum of hops in the range is as follows:

(13) $$\bar{H}=\frac{1}{N}\sum_{i=1}^{N}H_{i}$$

$P_{\alpha }$ is mainly related to the maximum power value of nodes. Compared with other cluster nodes, it is assumed that the CH power value is higher. With this in mind, the average power of network nodes is calculated as follows:

(14) $$\bar{P}=\frac{1}{N}\sum_{\alpha =1}^{N}P_{\alpha }$$

#### 4.1.2. Cluster Construction

After the CH is selected, the cluster is established through the neighbor nodes of the CH. In order to reduce the overhead of nodes in a cluster, the number of nodes in each cluster cannot exceed the upper limit β. When the nodes that do not belong to any cluster enter the transmission range of CH and the number of nodes in the cluster does not exceed β, the node is selected as CM by CH.

When a dynamic cluster moves from one RSU in a cooperative region to within range of another RSU, the request content cached in the previous RSU may be out of date, and the next RSU will not cache the requested content in advance for the upcoming cluster. Faced with this inefficient use of caching resources, we can use the mobility of the cluster to obtain the next RSU to arrive in the cluster and replace the cached content on the upcoming RSU in advance through cooperative transfer between RSUs. The created dynamic vehicle clusters can help replace cached content on RSUs, reducing content fetching latency and improving user experience.

### 4.2. Content Popularity Prediction Based on Temporal Convolutional Networks

In previous work, most studies have assumed by default that content popularity obeys a Zipf distribution [30,31,32]. In practice, due to the high-velocity mobility of vehicles and time-varying user demand, content popularity characteristics are difficult to capture in a timely manner and can only be predicted from historical information. Thus, this paper proposes a prediction method based on TCN. The TCN model consists of a content feature prediction module and a content popularity assessment module. After the content features are captured by the predictor module, the popularity assessment module will provide a weighted average of the past popularity data, thereby predicting the future popularity of the content, achieving a balance between long-term and short-term burst memory.

#### 4.2.1. Content Feature Predictor

**Figure 2:**
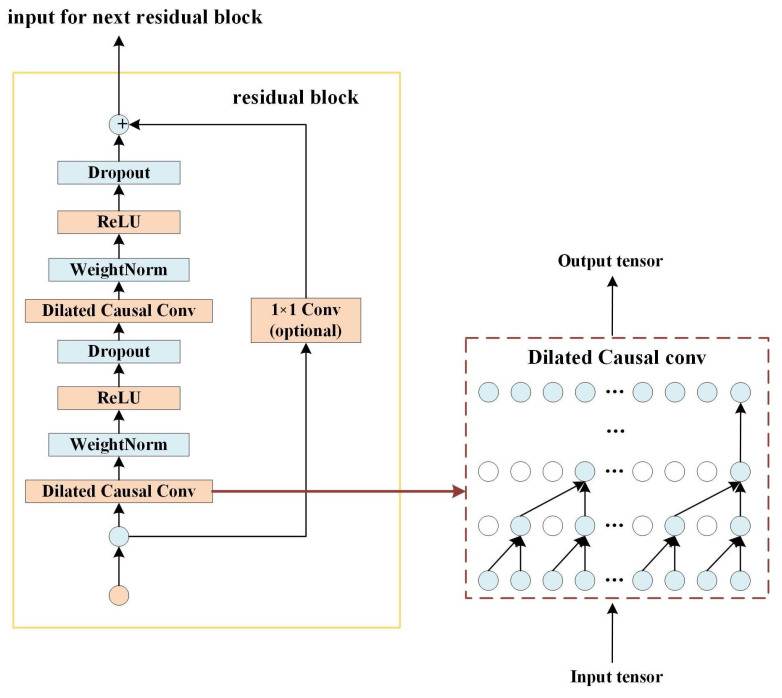
TCN prediction for caching.

The purpose of this module is to build input–output maps based on TCN, thereby predicting the popularity of future content through historical request information, to help THCS make effective caching decisions. Specifically, the input vector of TCN is a content popularity feature, and the expected output is a collection of content popularity features over the next *K* time periods. This article uses TCN to predict content popularity because it can transform the problem of content popularity prediction into a time series problem; moreover, compared with the commonly used recurrent neural network (RNN), TCN is based on a parallel architecture and takes up less memory. In addition, TCN can prevent gradient explosion and disappearance; the architecture is shown in Figure 2.

Specifically, the input vector of TCN is a content popularity feature, and the expected output is a collection of content popularity features over the next *K* time periods. TCN uses a one-dimensional full convolution network (FCN) structure for prediction, and due to the causal relationship between convolution operations, future information will not leak into the past. Next, we will explain the fusion process of TCN and convolution structure in detail.

Dilated causal convolution: In order to establish long-term memory, causal convolution can introduce dilated convolution to ensure that the convolutional receptive field can be increased without changing the number of parameters. The principle can be simply understood as that dilated convolution can generate some new kernels, and then use these kernels to perform ordinary convolution. For the filter $Y=\{y_{1},y_{2},\ldots ,y_{k}\}$, the dilated casual convolution $b_{t}$ of $V=\{v_{1},v_{2},\ldots ,v_{T}\}$ at $v_{t}$:

(15) $$b_{t}=\sum_{k-1}^{K}y_{k}v_{t-(K-t)d}$$

where *d* represents the dilated factor and *k* is the size of the filter.

Residual block: After normalizing the weight of the dilated causal convolutions each time, the ReLU function is used to increase the nonlinear relationship between layers, and then a dropout term is added to achieve regularization. In addition, in order to prevent the TCN output from being inconsistent with the length of *X*, a 1×1 convolution is performed before the output, so that the TCN can output in the desired dimension.

#### 4.2.2. Future Content Popularity Assessment

In order to achieve a balance between short-term and long-term memory in the popularity data ${\bar{P}}_{m,i,t+1}$ output at the next moment, we will perform a weighting operation between the historical popularity $P_{m,i,t}$ and the predicted short-term future popularity ${\hat{P}}_{m,i,T+1}$. The specific operations are as follows:

(16) $${\bar{P}}_{m,i,T+1}=\left(1-\lambda \right){\hat{P}}_{m,i,T+1}+\sum_{t=T-n+1}^{T}\lambda^{T-t+1}P_{m,i,t}$$

where λ(0<λ<1) is the weighted ratio between the new and historical data, and the larger λ represents the more important historical popularity; *n* represents the number of historical data we consider.

### 4.3. Hybrid Content Value (HCV)

The traditional popularity-based caching strategy is to improve cache performance by caching the most popular content in RSU. Most of them ignore the cooperation between RSUs, resulting in multiple RSU caches of the same content, which can easily cause cache redundancy. Moreover, if only popularity is considered without taking into account other factors that affect caching, overall performance will be affected.

Facing this problem, we propose a new metric named Hybrid Content Value. From the perspective of cooperation, HCV combines factors such as content popularity, content size, and transmission delay. When a user requests content $e_{i}$ from a cooperative RSU, the average delivery delay of the user-perceived request content $e_{i}$ is given by the following formula:

(17) $$\sum_{n=1}^{M}d_{r_{\varphi },r_{n},i}P_{n,i,t}$$

HCV $Z_{t}\left(r_{m},e_{i}\right)$ weights the value of content $e_{i}$ on $r_{m}$ for different situations.

Case 1: If RSU $r_{m}$ does not cache content $e_{i}$, the average delay of requesting content $e_{i}$ after $r_{m}$ caching content $e_{i}$ is as follows:

(18) $$\sum_{n=1}^{M}d_{r_{m},r_{n},i}p_{n,i,t}$$

where $x_{m,i}=0$, if $r_{m}$ caches content $e_{i}$, the benefit of HCV $Z_{t}\left(r_{m},e_{i}\right)$:

(19) $$\begin{array}{c} \begin{array}{cc} Z_{t}\left(r_{m},e_{i}\right) & =\sum_{n=1}^{M}d_{r_{\varphi },r_{n},i}P_{n,i,t}-\sum_{n=1}^{M}d_{r_{m},r_{n},i}P_{n,i,t}\\ \; & =\sum_{n=1}^{M}\max\left\{d_{r_{\varphi },r_{n},i}-d_{r_{m},r_{n},i},0\right\}P_{n,i,t} \end{array} \end{array}$$

Case 2: If RSU $r_{m}$ does cache content $e_{i}$, the average latency of user requests for content $e_{i}$ after $r_{m}$ removes content $e_{i}$ is as follows:

(20) $$\sum_{n=1}^{M}d_{r_{m},r_{n},i}p_{n,i,t}$$

where $x_{m,i}=1$, if $r_{m}$ removes content $e_{i}$, loss of HCV $Z_{t}\left(r_{m},e_{i}\right)$:

(21) $$\begin{array}{c} \begin{array}{cc} Z_{t}\left(r_{m},e_{i}\right) & =\sum_{n=1}^{M}d_{r_{m},r_{n},i}P_{n,i,t}-\sum_{n=1}^{M}d_{r_{\varphi },r_{n},i}P_{n,i,t}\\ \; & =\sum_{n=1}^{M}\max\left\{d_{r_{m},r_{n},i}-d_{r_{\varphi },r_{n},i},0\right\}P_{n,i,t} \end{array} \end{array}$$

### 4.4. Decision Making Based on Dynamic Planning

In order to obtain the overall optimal HCV, this paper will present the problem P2 on the basis of P1, which is expressed as follows:

(22) $$\begin{array}{c} P2:\max\sum_{m=1}^{M}\sum_{i=1}^{k}Z_{t}\left(r_{m},e_{i}\right)x_{m,i}\\ \;s.t.\;\sum_{i=1}^{F}x_{m,i}\;S_{i}\leq u_{m},\forall r_{n}\in R\\ x_{m,i}\in \left\{0,1\right\},\forall r_{n}\in R,\forall e_{i}\in E, \end{array}$$

For the proposed problem P2, we consider using the dynamic programming algorithm (DP) to divide the different stages of the cache to solve. P2 is described in detail as follows:

We use {st[1],…,st[i],…,st[k]} to represent the different stages, and the caching decisions for different stages are represented by $z_{i}$. $R_{i,j}$ represents the status of the st[i] phase, as follows:

(23) $$R_{i,j}=\max\sum_{k=1}^{i}Z_{t}\left(r_{m},e_{k}\right)W_{k}$$

(24) $$\;s.t.\;\max\sum_{k=1}^{i}W_{k}s_{k}+j\leq u_{m}$$

where whether or not to cache content $e_{k}$ in stage st[i] is determined by $W_{k}$.

When deciding on cache $e_{i}$ in the st[1] stage, THCS needs to check whether the current cache space can accommodate content $e_{i}$, the optimal solution of $R_{i,j}$ is as follows:

(25) $$\begin{array}{c} R_{i,j}=\left\{\begin{array}{cc} \max\left\{R_{i-1,j},R_{i-1,j-s_{i}}\right\}+Z_{t}\left[r_{m},e_{i}\right] & s_{i}\leq j\\ R_{i-1,j} & s_{i}>j \end{array}\right. \end{array}$$

When the current cache space is $s_{i}\leq j$, if the $r_{m}$ cache content $e_{i}$ cache space becomes $j-s_{i}$, $R_{i,j}$ becomes $R_{i-1,j-s_{i}}+Z_{t}\left[r_{m},e_{i}\right]$; otherwise, $R_{i,j}$ becomes $R_{i-1,j}$. When the remaining space $s_{i}\geq j$, $R_{i,j}$ becomes $R_{i-1,j}$. Based on this, the optimal decision process *Z* can be obtained. Algorithm 1 summarizes the dynamic programming algorithm.
**Algorithm 1** HCV-based dynamic planning caching algorithm.**Input:** Cache space $u_{m}$, Cache remaining space *j*, Total cache content *k*, $a_{i}=\left(e_{id_{i}},s_{id_{i}},r_{id_{i}}\right)$.**Output:** Decision *Z*.1:**for** i=1 to *k* **do**;2:    **for** j=1 to $u_{m}$ **do**;3:       **if** $a_{i-1,1}>j$ **then**;4:           $R_{i,j}=R_{i-1,j}$;5:       **else**6:           $R_{i,j}=\max\left\{R_{i-1,j},R_{i-1,j-a_{i-1,1}}+a_{i-1,2}\right\}$;7:       **end if**8:    **end for**9:**end for**10:Get cache parameters $\left(R,Z,k,u_{m}\right)$11:update *Z*


## 5. Experimental Results and Analysis

In this section, we compare the performance of the proposed THCS scheme with other benchmark schemes. Then, this article verifies the effectiveness of the model established in this article by comparing the prevalence prediction model established by TCN with classic prevalence prediction models.

### 5.1. Simulation Settings

This article considers setting up four RSUs in the cooperation area, and the cache capacity of each RSU is 256. Suppose there are 1000 video files in the network, the file size is a random number [33] in {1,3,5,7} and it follows the Zipf distribution [34] model with a coefficient of 0.55. We set the transmission delay of sending file *i* from the central server to RSU $r_{m}$ to be 10–20 ms, and the transmission delay between RSUs within the cooperation range is 2–4 ms, and the default delay of transmitting file *i* between RSUs is equal. In this paper, random search is used to set the parameters of the established TCN prediction model, which effectively avoids the curse of dimensionality. We set the parameters of the TCN prediction model by Random Search [35], which samples the search space instead of brute-forcing all possible parameter sets, thus avoiding the curse of dimensionality. The specific parameters are shown in Table 1 below:

### 5.2. Comparison of Algorithms and Indicators

LFU: This strategy prioritizes replacing the least frequently used content when storage space is low.

LRU: This strategy prioritizes replacing the least recently used content when storage space is low.

ECC [31]: This strategy uses the neural cooperative filtering algorithm to obtain popular content and then uses the greedy algorithm to optimize the caching strategy.

Distributed: This strategy caches a portion of top-ranked popular content and does not cache duplicate content, so distributed policies can cache more content items.

P_scheme [36]: This strategy establishes vehicle state and mobility models in the IoV network of ICN and performs active caching.

This article shows how THCS improves system performance through two metrics, namely, cache hit ratio (HR) and average content delivery latency (ADL).

HR represents the hit content as a percentage of the request content. Specifically, HR=B/G, where *G* represents the total number of requests received during period Δt, and *B* is the number of request hits.

ADL indicates the average delay of the request, expressed as:

(26) $$ADL=min\sum_{m=1}^{M}\sum_{i=1}^{F}P_{t}\left(r_{m},e_{i}\right)d_{r_{\varphi },r_{m},i}$$

### 5.3. Analysis of Results and Comparison of Performance

**Figure 3:**
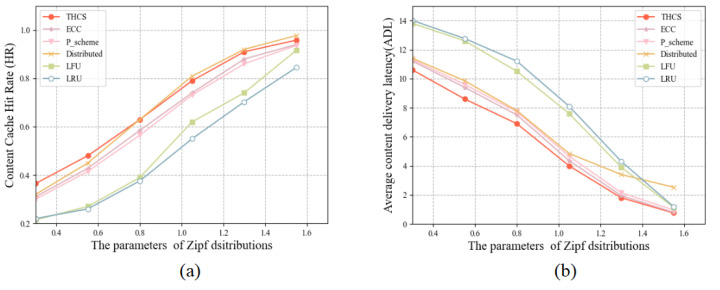
The relationship between cache performance and Zipf parameters: (**a**) HR for different Zipf parameters, (**b**) ADL for different Zipf parameters.

To investigate the relationship between content popularity and cache performance, we changed the content heat distribution by adjusting parameter β of the Zipf distribution. As shown in Figure 3, the cache performance improves as the parameters increase. This is because as the parameters β become higher, more cache space is allocated to the more popular content. Compared to the ECC strategy, the THCS strategy improved HR by 1–12% and reduced ADL by 5–12.5%.

**Figure 4:**
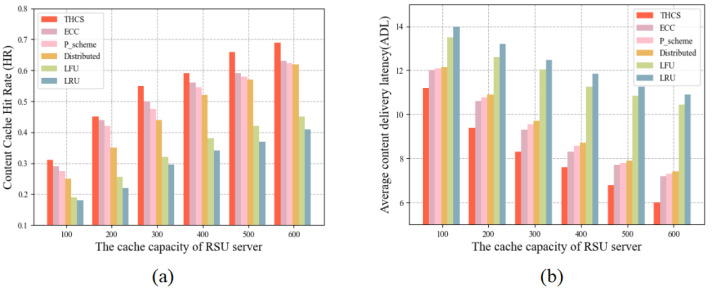
The relationship between cache capacity and cache performance: (**a**) HR for different cache capacity, (**b**) ADL for different cache capacity.

To investigate the effect of cache size on cache performance, we scaled the cache size of each RSU server from 100 to 600. As can be seen in Figure 4, cache performance improves as the cache size increases. The reason is that as the cache capacity increases, more content can be cached on the RSU servers and user requests are more likely to be available in the cooperation area rather than from remote servers. The THCS solution shows optimal performance in terms of both HR and ADL.

**Figure 5:**
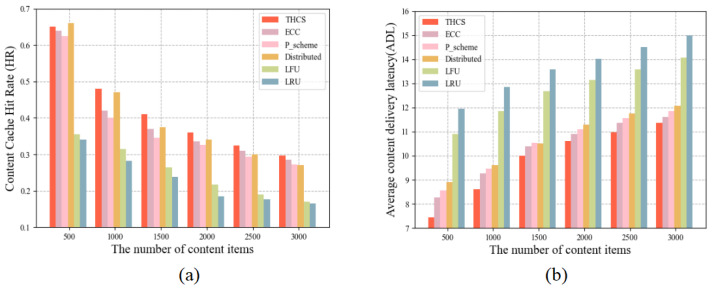
The relationship between cache performance and content quantity: (**a**) The influence of different content quantity on HR; (**b**) The influence of different content quantity on ADL.

To investigate the relationship between content count and cache performance, we limit the content count to the interval 500–3000. From Figure 5, we can see that content count is negatively correlated with cache performance, which is because more content items cause the RSU not to cache all content, so that more content cannot be fetched directly from the cooperation region. This paper proposes that the THCS policy continues to outperform other policies because the THCS policy considers the selection of cached content more comprehensively.

**Figure 6:**
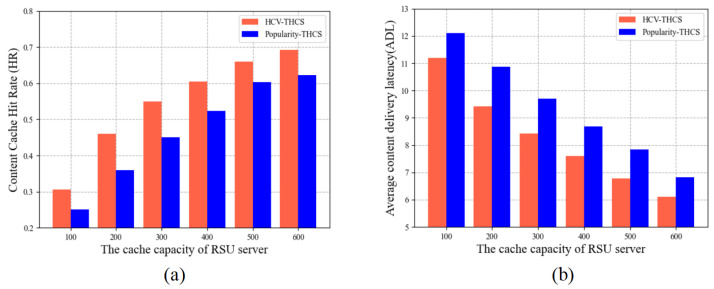
The relationship between cache capacity and cache performance: (**a**) HR with different cache capacity, (**b**) ADL with different cache capacity.

Figure 6 compares the traditional content popularity-based caching strategy with the caching strategy proposed in this paper that integrates HCV (denoted as Popularity-THCS and HCV-THCS). As shown in Figure, the trends of HR and ADL of the two approaches as the cache capacity increases, it can be seen that the HCV-THCS proposed in this paper exhibits superior performance, with an 8.6–16% increase in HR and a 7.3–14.3% decrease in ADL compared with the traditional scheme HCV-THCS.

## 6. Conclusions

In this paper, we propose the THCS strategy to improve cache performance. Firstly, through dynamic vehicle and cluster construction, we optimize the cache location. Then, we introduce the concept of HCV in cache content selection, combining different attributes to evaluate the cache value of the content. Finally, this paper formulates a caching decision based on dynamic programming to obtain a near-optimal caching solution. The experimental results show that our proposed scheme can cache more content closer to the requester. Simulation experiments and performance results show that THCS shows good performance in terms of cache hit rate and average content delivery delay. In future research, we will continue to investigate ways to reduce transmission latency and improve cache hit ratios in vehicle edge networks. Spatial prediction via neural graph networks or learning topological relationships between dynamic vehicles have been considered to further optimize caching strategies.

## Figures and Tables

**Table 1:** Simulation parameter table.

Parameter	Value
residual network depth	10
dilation interval	1, 2, 4, 8
kernel size	2
sliding window	2000
sliding step length	200
input length	20

## Data Availability

Not applicable.
